# Enhanced Vitamin D_3_ Adsorption Through Novel Hydrophobic Halloysite–Alginate Biopolymer Composites

**DOI:** 10.3390/polym17081083

**Published:** 2025-04-17

**Authors:** Mervenur Kirazoğlu, Birgül Benli

**Affiliations:** 1 Nano Science, and Nano Engineering M.Sc. Program 1, Graduate School, Istanbul Technical University, 34467 Istanbul, Türkiye; benli@itu.edu.tr; 2 Department of Mineral Processing Engineering 2, Faculty of Mines, Istanbul Technical University, 34467 Istanbul, Türkiye

**Keywords:** alginate-based delivery systems, halloysite nanotubes, vitamin D_3_ adsorption, controlled release, encapsulation efficiency, biopolymer nanocomposites, polymeric nanocarriers

## Abstract

This study presents a sustainable strategy to enhance polymer encapsulation, adsorption, and functional properties by chemically modifying sodium alginate with hydrophobic groups. Hydrophobic alginate derivatives were synthesized via a solvent-free method using hexadecyl trimethylammonium bromide, resulting in nanoparticles capable of effectively capturing non-polar compounds. To further improve compatibility within alginate-based biocomposites, halloysite nanotubes were modified through ball milling and surfactant-assisted treatments. The resulting nanocomposites (MBHA and MHHA) exhibited significantly enhanced adsorption and controlled release behavior, as confirmed by FTIR analysis of hexadecyl alginate ester conjugation. Vitamin D_3_ adsorption followed the Langmuir isotherm, with high correlation coefficients (R^2^ = 0.998 for MBHA and R^2^ = 0.991 for MHHA), indicating monolayer adsorption on a homogenous surface. Kinetic modeling revealed that the adsorption process adhered to a pseudo-second-order model (R^2^ = 0.9969 for MBHA and R^2^ = 0.999 for MHHA), suggesting that chemisorption was the dominant rate-controlling mechanism. These results demonstrate the critical role of surface modification in designing nano-engineered biopolymers with superior adsorption, stability, and release profiles, offering sustainable applications in medicine, agriculture, and environmental remediation.

## 1. Introduction

Polymeric nanocarriers have emerged as a transformative platform across various fields due to their biocompatibility and multifunctionality, offering significant advantages for the delivery of bioactive compounds [1,2,3]. By enhancing stability and enabling controlled release, these nanocarriers protect encapsulated agents from degradation and maintain therapeutic levels over time. Their nanoscale dimensions and high surface-area-to-volume ratio facilitate the traversal of biological barriers and improve overall cellular uptake. While they do not inherently provide targeted delivery, these properties can enhance the absorption and bioavailability of lipophilic compounds such as vitamin D_3_ in systemic applications.

**Figure 1:**
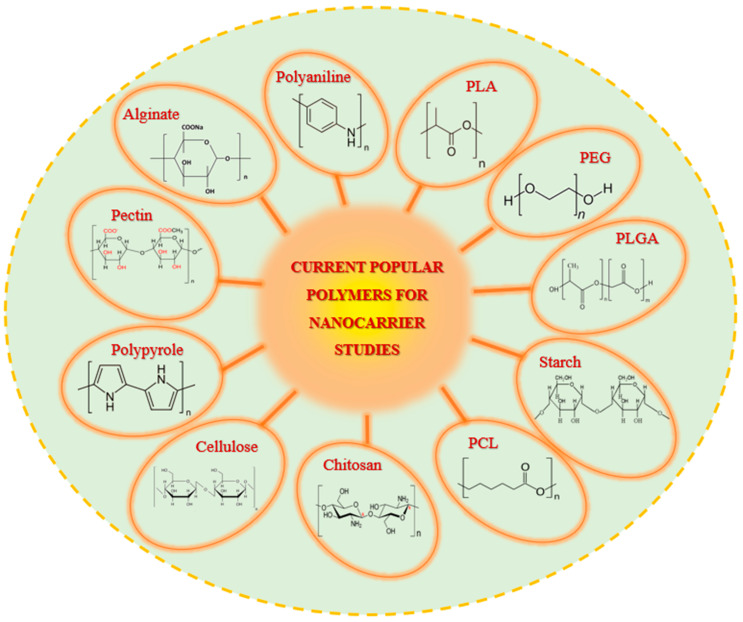
Current popular polymers for nanocarrier fabrication.

These nanocarriers can be tailored to suit diverse applications by utilizing biocompatible and biodegradable polymers, including polylactic acid (PLA), poly (lactic-co-glycolic acid) (PLGA), chitosan, alginate, and polycaprolactone (PCL) (Figure 1) [4]. Among these, alginate possesses several unique properties that distinguish it from other biopolymers. Notably, its pH sensitivity is highly beneficial in drug delivery systems, enabling controlled release in environments with varying pH levels, such as the stomach and intestines [5]. Alginate also rapidly forms hydrogels in the presence of divalent ions, such as calcium, facilitating the encapsulation and protection of therapeutic compounds [6,7]. Its high water-retention capacity, low irritation potential, and economic large-scale production further strengthen its appeal in biomedical and pharmaceutical applications [8,9].

Alginate is a naturally occurring anionic polysaccharide primarily extracted from brown seaweed, composed mainly of mannuronic and guluronic acid units. Its excellent biocompatibility, gel-forming ability, and pH sensitivity make it particularly valuable for drug delivery and controlled-release systems. However, issues such as burst release and poor encapsulation efficiency limit its practical performance [10]. To address these limitations, researchers have explored modifying alginate with surfactants and incorporating various fillers such as halloysite nanotubes (HNTs) [11]. Surfactants improve gel uniformity and stability, reduce burst effects, and increase encapsulation efficiency. HNTs contribute by acting as diffusion barriers, and providing a high surface area with abundant binding sites. Their nanotubular morphology enables sustained release and enhanced adsorption of bioactive components, which are gradually released as the alginate gel matrix degrades [12,13].

Halloysite, a naturally occurring aluminosilicate mineral, presents a tubular nanostructure that provides advantages in drug delivery and molecular encapsulation applications [14]. Its hollow tubular morphology facilitates uniform payload distribution and protection against environmental degradation under acidic, alkaline, or enzymatic conditions [15,16]. Halloysite nanotubes are also mechanically robust, which contributes to the overall physical stability of the nanocarriers. This robustness, combined with improved surface compatibility through hydrophobic modifications, may help reduce sedimentation and aggregation during long-term storage or transportation, as supported in previous nanocomposite systems [17]. Furthermore, the surface of halloysite is highly amenable to functional modifications, enabling improved stability in biological environments [18]. Hydrophobic surface modification may help reduce interactions with water and improve interfacial compatibility within the alginate matrix, contributing to better dispersion and structural stability. However, in aqueous systems, hydrophobic surfaces alone are not sufficient to prevent aggregation, and additional stabilization strategies are often necessary [19]. These modifications can increase water resistance, modulate biological interactions, and facilitate greater non-toxicity, thereby improving the safety and long-term usability of the nanocarrier systems [20].

One-pot hydrophobization is a promising strategy for enhancing nanocarrier stability in aqueous and biological systems [21]. By increasing water resistance, reducing aggregation, and improving interactions with cell membranes, hydrophobic modifications can enhance the bioavailability, targeting accuracy, and shelf life of nanocarriers [22,23]. Despite the clear benefits, the literature on biopolymer hydrophobization, especially involving alginate–HNT systems, remains limited [24]. To address this research gap, the present study explores the encapsulation and adsorption potential of hydrophobically modified alginate–HNT composites for vitamin D_3_ delivery. This integrated approach offers enhanced stability and bioavailability, making it attractive for food fortification and nutraceutical applications [25,26].

Vitamin D_3_ is a vital micronutrient essential for calcium and phosphorus metabolism and bone health [27]. However, its limited presence in natural food sources has led to widespread deficiency, associated with increased risk of infections, cancer, and immune system dysfunction [28,29]. Effective supplementation and dietary fortification strategies are therefore necessary to address this global health issue.

Vitamin D_3_’s lipophilic nature and poor solubility pose challenges for its incorporation in functional foods and supplements [30,31]. Conventional delivery strategies are often insufficient to protect it from degradation or ensure efficient absorption. Nanocarriers present a promising strategy by enhancing the solubility and bioavailability of vitamin D_3_ through encapsulation, thereby ensuring its stability and enabling controlled release for improved absorption [32]. This approach supports adequate vitamin D levels and increases the nutritional value of fortified products, adding substantial value to the food and nutraceutical industries [33,34].

Although vitamin D_3_ is typically absorbed through micelle formation with bile salts, alternative absorption routes also exist, especially when the compound is stabilized and solubilized using delivery systems [35,36]. Our composite system does not directly promote micelle formation but improves vitamin D_3_ bioavailability through multiple mechanisms. The hydrophobically modified alginate creates a favorable environment for the lipophilic vitamin, enabling efficient encapsulation. Halloysite nanotubes contribute by offering a high surface area and protection against degradation. Moreover, the composite supports sustained release under physiological conditions, increasing the residence time in the intestine. These combined effects enhance absorption and efficacy, even without classical micelle-based transport.

This study aims to improve the encapsulation and adsorption capabilities of alginate-based delivery systems. A hydrophobic alginate derivative, termed hexadecyl alginate ester, was synthesized using hexadecyl trimethylammonium bromide (HTAB) under solvent-free conditions. HTAB, belonging to the quaternary ammonium salt group, was selected as a cationic surfactant due to its well-characterized surface activity, making it suitable for model studies on hydrophobic modification. It is commonly used in antimicrobial, emulsifying, and surface modification applications. In this study, HTAB was selected as a model hydrophobic modifier to investigate the effects of one-pot hydrophobization on the encapsulation and adsorption behavior of alginate-based nanocarriers. Its well-known molecular behavior provides a controlled platform for evaluating hydrophobically modified systems, prior to the incorporation of more sustainable, bio-based alternatives. The self-assembling nanoparticles derived by hexadecyl alginate ester provide a stable matrix for the incorporation of hydrophobic nutraceuticals. In parallel, halloysite nanotubes were functionalized with surfactants to enhance their compatibility with alginate-based biocomposites, thereby improving encapsulation efficiency and enabling controlled-release functionality.

## 2. Materials and Methods

### 2.1. Materials

Halloysite clay nanotubes were supplied by ESAN (Eczacıbaşı Industrial Raw Materials Co., Istanbul, Türkiye). Sodium alginate was obtained from Sigma-Aldrich (Sigma-Aldrich Corporation, St. Louis, MI, USA). Formic acid (98%) was purchased from Aksin Kimya (Aksin Kimya Sanayi ve Ticaret A.Ş, İstanbul, Türkiye). Hexadecyltrimethylammonium bromide (HTAB) was obtained from Merck (Merck KGaA, Darmstadt, Germany). Methanol (99.99%) and ethanol (95%) were obtained from EMSURE (Merck KGaA, Darmstadt, Germany). Poly(diallyldimethylammonium chloride) solution (PDDA) and silane were also obtained from Sigma-Aldrich (Sigma-Aldrich Corporation, St. Louis, MI, USA). Deionized water was used throughout all experiments.

### 2.2. Preparation of Hexadecyl Alginate Ester

Hydrophobic alginates were synthesized by reacting sodium alginate with HTAB via esterification in the presence of formic acid, adapted from a previous study [37]. For the formylation step, 1 g of sodium alginate was mixed with 10 mL of formic acid and stirred for 10 min at 25 °C. Subsequently, 8 mL of HTAB solution was added dropwise. The mixture was heated to 50 °C for 20 min. The reaction was stopped by adding 100 mL of 95% ethanol, followed by filtration to collect the solid phase. The residue was repeatedly washed with 95% ethanol and vacuum-dried at room temperature [38].

### 2.3. Mechanical Activation of HNT Nanotubes via Ball Milling

**Figure 2:**
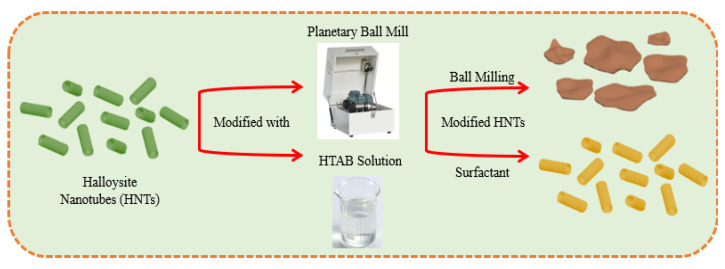
Schema of modified HNTs via two approaches.

Initially, HNT nanotubes were dried at 67 °C for one hour to remove moisture, which can interfere with hydrophobic reactions. Drying ensures optimal surface conditions for binding hydrophobic agents [39]. The dried HNTs were ground for 0.5, 1, 2, 3, and 4 h at room temperature using a planetary ball mill (OPT-SFM3, OPTOSENSE LLC, Winter Park, FL, USA) at 300 rpm (Figure 2). A 10 mL ball mill jar filled with zirconia beads (1:10 *w*/*w*) received 1 g of HNTs. Heat and shear forces generated during milling activated the jar walls [40]. Typically, zirconia beads 10–20 times the weight of the HNTs are used, but excessive beads may hinder homogeneity. Optimization was applied to achieve efficient milling [41,42]. The resulting composite was designated MBHA (ball-milled halloysite + hydrophobic alginate).

### 2.4. Surfactant Treatment of HNT Nanotubes

Before treatment, HNTs were dried at 110 °C for 3 h [43]. An aqueous surfactant solution was prepared by dissolving 4 g of HTAB in 500 mL of a 1:1 *v*/*v* water–ethanol mixture. Then, 8 g of HNTs was added, and the dispersion was stirred for 48 h. The functionalized material was recovered by centrifugation at 700 rpm for 40 min, washed with distilled water to remove excess surfactant, and dried at 80 °C for 7 days [44]. The resulting composite was named MHHA (HTAB-modified halloysite + hydrophobic alginate).

### 2.5. Hydrophobic Halloysite–Alginate Biocomposites

Vitamin D_3_ was extracted from an oral solution (1 mL) using 1 mL of methanol as a solvent in a standard liquid–liquid extraction procedure. According to label claims, the solution contains 300.000 IU (7.5 mg/mL) of vitamin D_3_, equivalent to 1.95 × 10^−2^ M (MW: 384.65 g/mol). The extraction was performed in the dark. Hydrophobically modified HNTs were then mixed with the vitamin D_3_ solution under constant stirring to achieve homogeneous dispersion while minimizing degradation under light-sensitive conditions [45].

### 2.6. Adsorption Experiments

Batch adsorption experiments were conducted to evaluate the vitamin D_3_ adsorption efficiency of the biocomposites. The percentage of adsorption was calculated based on the concentration difference between the initial and equilibrium states using Equation (1):

(1) $$\mathrm{Adsorption}\;(\%)=[\frac{C_{0}-C_{t}}{C_{t}}]\;*\;100$$

where C_0_ is the initial vitamin D_3_ concentration (ng/mL), and C_t_ is the concentration at time t (ng/mL).

Each 20 mL glass vial received 0.1 g of biocomposite and 6 mL of vitamin D_3_ solution at varying concentrations (53.576, 42.456, 37.503, 18.751, and 9.876 ng/mL). Vials were sealed and shaken at 200 rpm for 1 h, then centrifuged at 3000 rpm for 5 min. Supernatants were filtered (0.45 μm PTFE) and analyzed using UV-Vis spectrophotometry at 265 nm. The amounts of vitamin D_3_ adsorbed (q_e_, mg/g) were calculated using Equation (2):

(2) $$q_{e}=\frac{V}{m}{(C}_{0}-C_{e})$$

where V is the volume (mL), m is the adsorbent mass (g), C_e_ is the equilibrium concentration (mg/mL), and C_0_ is the initial concentration. According to the adsorption mechanism of vitamin D_3_ on the surface of the hydrophobic halloysite–alginate biocomposite, commonly used adsorption isotherms such as Langmuir, Freundlich, Redlich–Peterson, Sips, and Toth were used [46].

The Langmuir isotherm assumes monolayer adsorption on a homogeneous surface, where adsorption sites are energetically identical and each molecule has equal activation energy and equal affinity. Once a molecule occupies a site, no further adsorption can occur at that location. This model also assumes that the adsorbent has a finite adsorption capacity [47]. The Langmuir equation is given in Equation (3):

(3) $$q_{e}=\frac{q_{ml}K_{L}C_{e}}{1+K_{L}C_{e}}$$

where q_ml_ is the maximum adsorption capacity (mg/g), and K_L_ is the Langmuir constant (L/mg).

The Freundlich isotherm is an empirical model used to describe reversible and non-ideal adsorption on heterogeneous surfaces, where adsorption sites have varying energies. It can account for multilayer adsorption and uneven distribution of adsorption heat across the surface [48]. The Freundlich equation is given in Equation (4):

(4) $$q_{e}=K_{f}\;*\;{Ce}^{1/nf}$$

Here, K_f_ is the Freundlich constant (L/g) and n_f_^−1^ represents adsorption density. The model is suitable if 0 < n_f_^−1^ < 1. Values of n_f_^−1^ > 1 indicate unfavorable adsorption, while a value of 1 implies irreversible adsorption.

The Sips isotherm, also known as the Freundlich–Langmuir isotherm, is applied to predict adsorption on heterogeneous surfaces. It combines both the Langmuir and Freundlich models to overcome the limitations of the continuous adsorption increase in the Freundlich model. At low concentrations, it behaves like the Freundlich isotherm, while at high concentrations, it resembles the Langmuir isotherm [49]. The Sips equation is given in Equation (5):

(5) $$q_{e}=\frac{q_{ms}a_{s}{Ce}^{ns}}{1+a_{s}{Ce}^{ns}}$$

where q_ms_ is the maximum adsorption capacity (mg/g), a_S_ is the Sips equilibrium constant (L/g), and n_S_ is the Sips model exponent.

The Redlich–Peterson isotherm is a three-parameter model that combines the characteristics of the Langmuir and Freundlich isotherms. It has a linear relationship in the numerator and an exponential term in the denominator, making it applicable to both homogeneous and heterogeneous systems [50]. The equation is shown in Equation (6):

(6) $$q_{e}=\frac{K_{R}C_{e}}{{1+a}_{R}C_{e}^{g}}$$

where K_R_ is the Redlich–Peterson constant (L/g), a_R_ is the constant (1/mg), and g is the exponent (0 < g < 1).

The Toth isotherm is a modified form of the Langmuir model designed to minimize errors between experimental and theoretical values. It is suitable for heterogeneous systems at both low and high concentration levels. It assumes an asymmetric, quasi-Gaussian distribution of adsorption energy, where most regions have energy levels below the average [51]. The Toth equation is provided in Equation (7):

(7) $$q_{e}=\frac{q_{mt}C_{e}}{{{(K}_{T}+C_{e}^{tn})}^{1/tn}}$$

Here, q_mt_ is the maximum adsorption (mg/g), K_T_ is the Toth isotherm constant (L/g), and t_n_ is a constant between 0 and 1. When t_n_ = 1, the equation reduces to the Langmuir form.

### 2.7. Statistical Evaluation of Adsorption Isotherm Models

To ensure a statistically robust comparison of adsorption isotherm models, several goodness-of-fit metrics were employed beyond traditional R^2^ values. These included the Residual Sum of Squares (RSS), Akaike Information Criterion (AIC), Bayesian Information Criterion (BIC), and chi-square (χ^2^) goodness-of-fit test. Each parameter provides a distinct perspective on model performance by balancing accuracy and complexity. Lower values of AIC, BIC, and χ^2^ are indicative of a better-fitting model.

All statistical analyses were conducted using Minitab^®^ software (Minitab16, LLC, State College, PA, USA) under an institutional license. The nonlinear regression fitting of the Langmuir and Freundlich models was performed using the built-in regression tools, and results were verified with Python 3.13.1 software (Python Software Foundation, Wilmington, DE, USA), based calculations and Microsoft Excel, yielding highly consistent results.

### 2.8. Desorption Experiments

Phosphate-buffered saline (PBS) at pH 7.4 was used to simulate intestinal fluid during the desorption experiments. Biocomposite samples (0.1 g) containing adsorbed vitamin D_3_ solutions (42 ng/mL) were homogenized and evenly distributed onto glass plates (surface area ~4 cm^2^) using a spin coater at 2000 rpm for 10 min. This coating process was repeated ten times per sample to ensure sufficient surface coverage. The films were then dried in an oven at 50 °C. Subsequently, they were immersed in 20 mL of PBS (pH 7.4), and desorption was monitored by measuring absorbance at 265 nm.

### 2.9. Characterization Studies

Characterization of the prepared biocomposites was carried out through multiple analytical techniques. A UV-Vis spectrophotometer (Shimadzu UV-1208, Shimadzu Corporation, Kyoto, Japan) was employed to analyze optical properties at specific wavelengths. Structural features were determined using FT-IR spectroscopy (Nicolet 6700, Thermo Fisher Scientific, Waltham, MA, USA) and X-ray diffraction (XRD; Bruker D8 Advance, Bruker AXS GmbH, Karlsruhe, Baden-Württemberg, Germany). FT-IR analysis confirmed chemical modifications and identified characteristic functional properties in the biocomposite structure.

Surface wettability was assessed using the sessile drop method with a Dataphysics OCA-15 contact angle analyzer. Surface charge (zeta potential) measurements were obtained via a Malvern Zetasizer (Malvern Panalytical Ltd., Worcestershire, UK). Morphological analysis of both unmodified and modified materials was conducted using scanning electron microscopy (SEM; Thermo Fisher Scientific, Waltham, MA, USA). The obtained results confirmed the successful fabrication of biocomposites, emphasizing the importance of optimizing the synthesis process to achieve enhanced performance.

## 3. Results and Discussion

### 3.1. Structural Characterization of Biocomposites

Important evidence for understanding the bonding in composite structures is provided by FTIR studies. Intermolecular interactions at the molecular level affect the width, intensity, and peak positions of spectral bands [52]. FTIR spectra were obtained for pure halloysite nanotubes, pure alginate, hydrophobic halloysite (modified via ball milling and HTAB surfactant treatment), modified alginate, and two different biopolymer ratios in the range of 4000–625 cm^−1^. In the first step of the synthesis method, sodium alginate was dissolved in formic acid, resulting in the in situ formation of formate esters. These esters reduce hydrogen bonding between alginate chains, exposing more hydroxyl groups. HTAB, a highly reactive reagent, was then used to create hexadecyl alginate esters (HAEs) with a high degree of substitution by reacting with the accessible hydroxyl groups. Since formate esters are inherently unstable, they hydrolyze at the end of the reaction, leaving pure HTAB esters of alginate as the final product.

**Figure 3:**
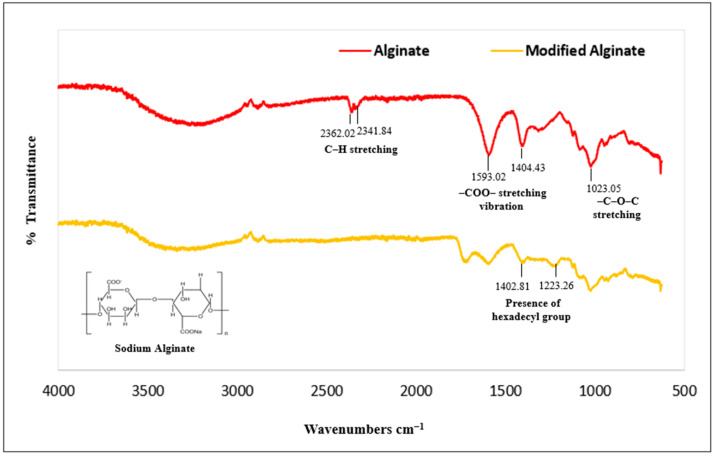
FTIR spectra comparing pure and modified samples, showing characteristic ester and alkyl group peaks.

The FTIR spectra of pure alginate and HAE, as shown in Figure 3, suggest the presence of ester bonds and hexadecyl groups. The reduced peak at 1593.02 cm^−1^ corresponds to asymmetric stretching of carboxyl groups, while the increased peak at 1722.69 cm^−1^ indicates C=O stretching vibration, confirming the presence of ester bonds [53]. Increased peaks at 1223.26 cm^−1^ and 1402.81 cm^−1^ indicate the presence of hexadecyl groups. The peak at 1223.26 cm^−1^ corresponds to C-C stretching vibration [40], and the peak at 1402.81 cm^−1^ corresponds to CH_2_ deformation vibration. Our FTIR spectrum analysis is consistent with the literature [41,54,55], suggesting that the materials share similar chemical structures, and that the nanoparticle formulation was accurately executed. We further evaluated the potential of nanoparticles for achieving sustained release of vitamin D_3_.

**Figure 4:**
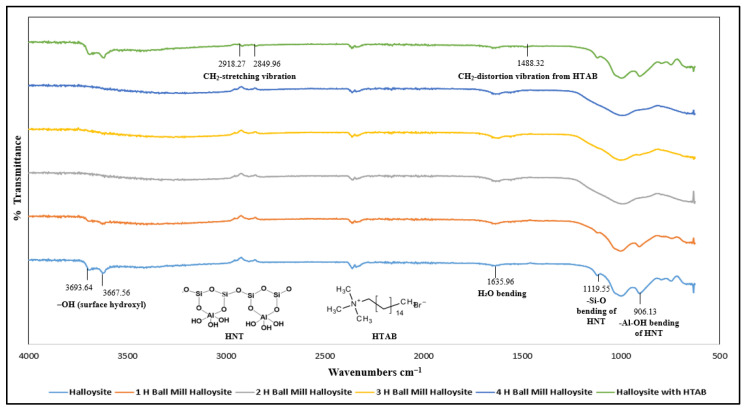
FTIR analysis of pure and modified HNTs, showing changes in the OH-group and CH₂ vibration regions.

Compared with unmodified HNTs, modified HNTs do not show significant changes in the 3500–3700 cm^−1^ range in Figure 4. This suggests that HTAB is not coated on the OH-groups inside the lumen or interlayer regions of the nanotubes [56]. Newly formed bands at 2918.27 cm^−1^ and 2849.96 cm^−1^ were attributed to asymmetric and symmetric CH_2_-stretching vibrations, respectively, while the band at 1488.32 cm^−1^ was attributed to CH_2_-distortion vibration from HTAB [57]. Ball milling of halloysite nanotubes for 1, 2, 3, and 4 h resulted in noticeable changes in the 3500–3700 cm^−1^ region, indicating modification of OH-groups within the inner space and interlayer of the nanotubes, as shown in Figure 4. Peaks at 3693.64 cm^−1^ and 3667.56 cm^−1^ disappeared after 1 h of ball milling, indicating structural changes in both surface and interior regions of the nanotubes.

**Figure 5:**
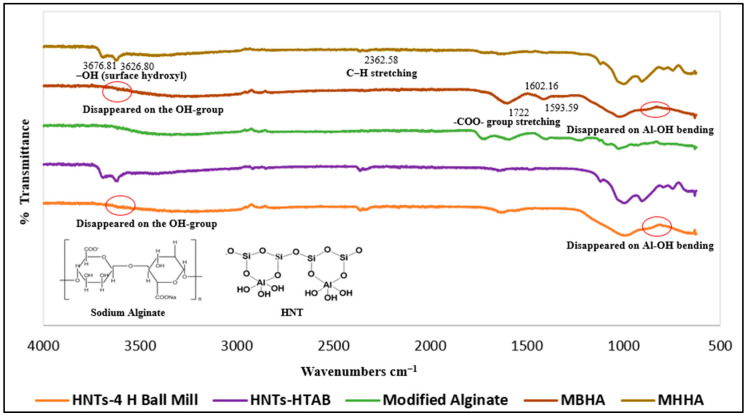
FTIR spectra of final biocomposites (MBHA, MHHA) showing the incorporation of modified halloysite and alginate components.

Biocomposites exhibited the characteristic peaks of both alginate and clay. The incorporation of modified HNTs into modified alginate did not lead to noticeable changes in peak positions or the emergence of new peaks, as shown in Figure 5.

**Figure 6:**
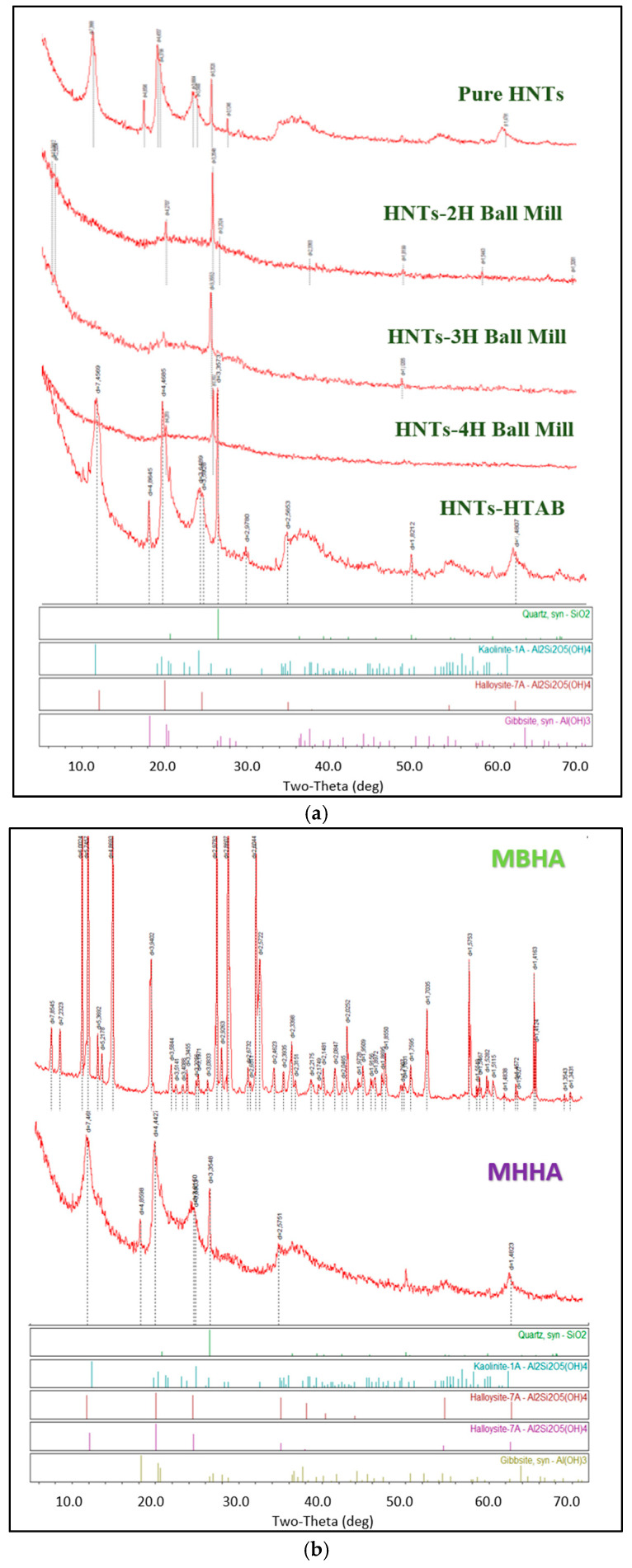
XRD patterns of modified samples and final biocomposites: (**a**) pure and modified HNTs; (**b**) MBHA and MHHA.

XRD analysis of pure HNTs revealed significant amounts of halloysite and kaolinite minerals, consistent with their natural origin (Figure 6a). This study involved modifying HNTs via HTAB surfactant treatment and mechanical activation through ball milling. While ball milling disrupted the tubular morphology and crystalline structure of pure HNTs, resulting in a silica-like structure, HTAB modification preserved the tubular and cage-like structure, with no new diffraction peaks observed. This indicates a successful surface modification without altering the fundamental characteristics of the HNTs. Additionally, XRD results of the biocomposites revealed structural differences: MBHA exhibited a silica-like amorphous structure, whereas MHHA retained its crystalline form with clear halloysite peaks (Figure 6b). These results highlight the versatility of HNTs in various applications and the influence of modification routes on structural outcomes.

**Figure 7:**
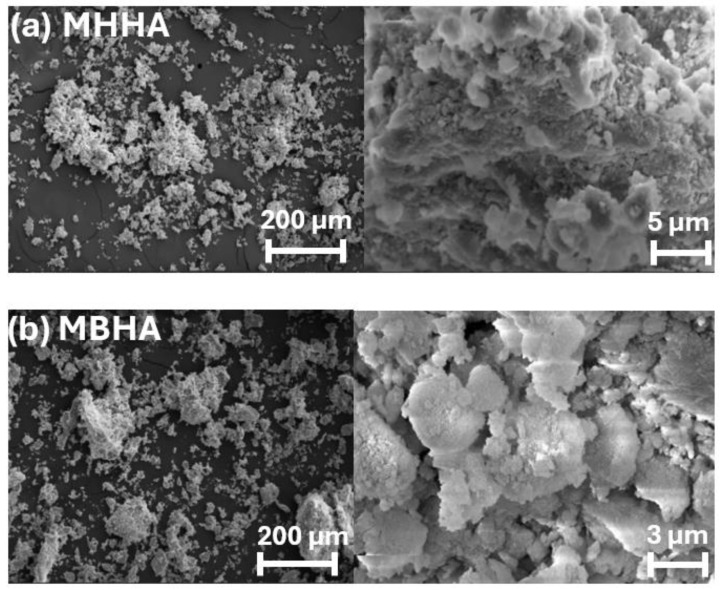
SEM images of pure and modified HNTs, and final biocomposites MBHA and MHHA, showing morphological differences.

The surface morphology of the synthesized samples was evaluated using scanning electron microscopy (SEM), as shown in Figure 7a,b. Pure halloysite nanotubes (HNTs) exhibited a uniform cylindrical shape with an average diameter of approximately 214.1 nm and a length of 543.7 nm, displaying homogeneous dispersion. In contrast, HTAB-modified HNTs subjected to 200 min of ball milling showed increased aggregation and partial disruption of the tubular structure due to mechanical stress.

Ball milling reduced the particle size of HNTs, enhancing contact between particles by breaking apart aggregates; however, it also compromised the integrity of the fibrous morphology. Biocomposites prepared using ball-milled halloysite and modified alginate displayed a more uniformly dispersed microstructure. In comparison, biocomposites formed with HTAB-modified halloysite and modified alginate exhibited more pronounced aggregation due to intensified surface interactions. These results highlight the critical role of surface modification strategies in shaping the microstructural features of the biocomposites.

### 3.2. Adsorption Studies of Vitamin D_3_ Solutions on Biocomposites

Adsorption experiments were conducted to identify the most efficient biocomposite for vitamin D_3_ retention. Concentrations were determined using calibration curves generated in this study, with each experiment repeated in triplicate.

**Figure 8:**
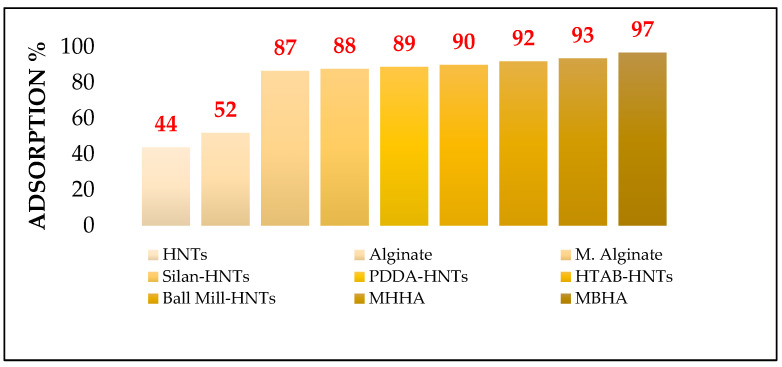
Comparison of vitamin D_3_ adsorption efficiency between modified and unmodified biocomposite samples.

As shown in Figure 8, MBHA (96.64%) and MHHA (93.45%) demonstrated high adsorption efficiencies, confirming their superior performance compared to pure alginate or HNT alone. The combination of alginate and modified HNTs produced a synergistic effect typical of compatible biocomposite systems, as supported by prior studies [40]. These results highlight the significance of surfactant-mediated surface modifications, particularly with HTAB, in enhancing adsorption capacity. The performance of MBHA and MHHA indicates strong potential for applications in nutrient delivery systems or environmental remediation, where high-efficiency adsorption is required.

While Figure 8 illustrates the relative adsorption efficiency among the prepared formulations, absolute adsorption capacity is a more reliable metric for evaluating true performance. To provide a broader context, Table 1 compares the adsorption capacity of our system with other vitamin D_3_ adsorbents reported in the literature. Although the capacity of the current system (3.0 mg/g) is moderate compared to some advanced sorbents, it offers significant advantages in terms of biocompatibility, biodegradability, and application in food and nutraceutical systems.

### 3.3. Hydrophobic Performance of Biocomposites

**Figure 9:**
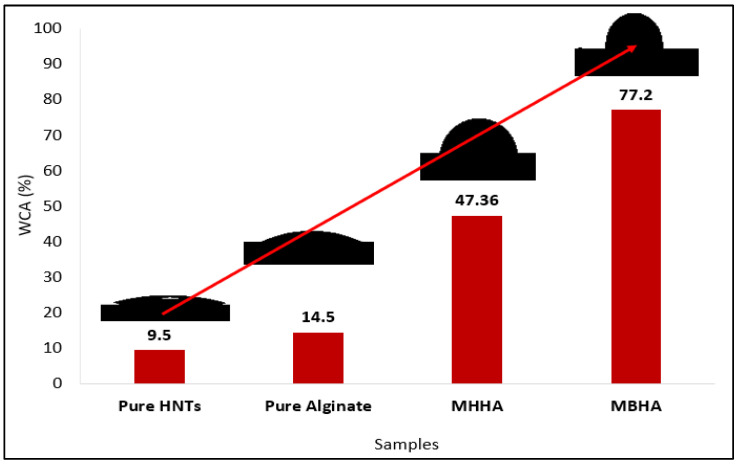
Contact angle values demonstrating the increased hydrophobicity of MHHA and MBHA biocomposites after surface modification.

Figure 9 illustrates the improved hydrophobicity of the biocomposites following surface modifications. The contact angle increased from 9.5° ± 0.8 (pure HNTs) to 77.2° ± 0.4 (MBHA), demonstrating successful surface functionalization. The significant increase in contact angle, especially for MHHA and MBHA, confirms the hydrophobic nature of the modified composites, making them suitable for water-resistant applications.

### 3.4. Adsorption Isotherm Studies

**Figure 10:**
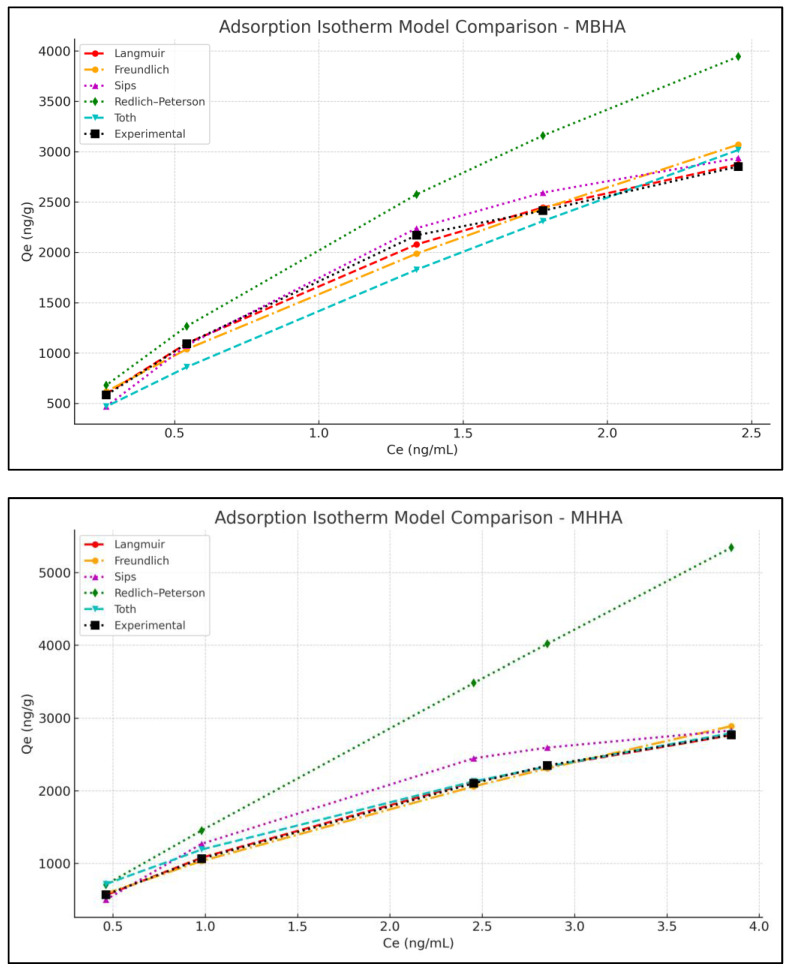
Adsorption isotherms of vitamin D_3_ at varying concentrations on MBHA and MHHA biocomposites.

Regression analyses were conducted using the Langmuir, Freundlich, Sips, Redlich–Peterson, and Toth isotherm models, with results presented in Table 2 and Figure 10. Among these, the Langmuir model best fit the experimental data for both MBHA and MHHA, suggesting monolayer adsorption on homogeneous surfaces. This adsorption behavior is advantageous for vitamin encapsulation, as it supports uniform distribution, stronger binding interactions, and potentially enhanced bioavailability.

According to the statistical evaluation, the Langmuir isotherm model provided an excellent fit for both MBHA and MHHA composites. Therefore, these biocomposites were compared based on their Langmuir parameters to assess adsorption efficiency (Table 3).

The model fitting was supported by multiple statistical indicators including R^2^, the Residual Sum of Squares (RSS), the Akaike Information Criterion (AIC), the Bayesian Information Criterion (BIC), and chi-square (χ^2^). MHHA exhibited a slightly higher maximum adsorption capacity (qmax = 5935.3 ng/g) than MBHA (qmax = 5318.1 ng/g), along with superior statistical fits—R^2^ = 0.9980, RSS = 2071.1, AIC = 47.89, and χ^2^ = 0.92—all lower than the respective MBHA values (R^2^ = 0.9916, RSS = 5632.4, AIC = 52.30, and χ^2^ = 2.39).

These results confirm the validity of a monolayer adsorption mechanism on homogeneous surfaces and highlight the enhanced adsorption capacity and surface interaction of MHHA compared to MBHA. The superior performance of MHHA underscores the effectiveness of surfactant-based surface modifications and demonstrates the potential of such biocomposites in controlled delivery applications, especially in biomedical systems.

### 3.5. Adsorption Kinetics Studies

Desorption-based release studies revealed that the adsorption process followed a second-order kinetic model. Kinetic data were obtained in PBS (pH 7.4), a buffer solution widely used to simulate intestinal fluid. This approach allowed the release profile of vitamin D_3_ to reflect behavior under physiological conditions, enhancing the relevance of the model for potential in vivo applications, supporting the applicability of the biocomposites in biomedical delivery systems. The adsorption kinetics of vitamin D_3_ on the biocomposites were best described by the pseudo-second-order kinetic model proposed by Ho and McKay (1999) [61], as shown in Table 4. The pseudo-second-order kinetic model fit suggests that vitamin D_3_ release from the composite occurs through chemical interactions rather than physical adsorption. The controlled release profile implies that these composites can deliver vitamin D_3_ sustainably, which is particularly advantageous for biomedical applications.

### 3.6. Desorption Studies

**Figure 11:**
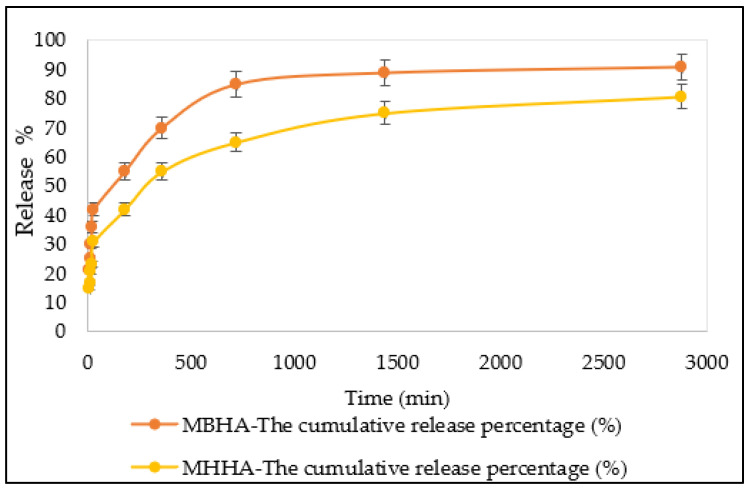
In vitro cumulative release profiles of vitamin D_3_ from MBHA and MHHA composites in PBS (pH 7.4) over time. Error bars represent ± standard deviation (n = 3).

Desorption studies were conducted in phosphate-buffered saline (PBS, pH 7.4) to simulate intestinal fluid and evaluate the release behavior of vitamin D_3_ from the biocomposites. An initial burst release was observed, with 42% of vitamin D_3_ released from MBHA and 30.8% from MHHA within the first 30 min. This was followed by a slower, sustained release phase, culminating in a total release of 48% from MBHA and 50% from MHHA over 48 h in Figure 11.

These results indicate that both biocomposites are capable of prolonged release under physiologically relevant conditions. Notably, MHHA exhibited a more controlled and gradual release profile, likely due to its preserved tubular structure and enhanced surface hydrophobicity. The vitamin D_3_ affixed to the biocomposites’ cores may be gradually released into PBS at pH 7.4, simulating a prolonged delivery behavior within gastrointestinal fluid. This controlled release behavior reinforces the potential of surface-engineered alginate–halloysite composites for applications in targeted nutrient delivery and sustained-release biomedical systems.

## 4. Conclusions

In this study, novel alginate-based biocomposites incorporating hydrophobic halloysite were successfully synthesized and evaluated as nanocarriers for the adsorption and controlled release of vitamin D_3_. Structural analyses confirmed the effective chemical modification of alginate via hexadecyl ester formation and the surface engineering of halloysite through surfactant-assisted and mechanical activation. These modifications yielded composites with enhanced hydrophobicity, improved dispersion, and robust morphological integrity.

Adsorption studies revealed that the Langmuir isotherm provided the best fit for both composites, indicating monolayer adsorption on a homogeneous surface. MHHA exhibited superior performance with a higher adsorption capacity (q_max_ = 5935.3 ng/g), stronger statistical agreement (R^2^ = 0.9980), and more sustained release behavior. Desorption studies in PBS (pH 7.4) confirmed prolonged vitamin D_3_ release over 48 h, closely simulating intestinal conditions.

The use of halloysite, a naturally occurring clay mineral, offered additional advantages in this study. Its tubular morphology, large surface area, and biocompatibility made it an ideal nanocarrier platform. Importantly, the halloysite used was a natural clay, applied directly after particle size reduction via ball milling, eliminating the need for complex synthetic processing. This enhances both the ecological and practical value of the system, supporting the development of sustainable material solutions.

These nanocarriers open up numerous applications across both the pharmaceutical and food industries. In the pharmaceutical field, nano-engineered systems can improve therapeutic efficacy through targeted and controlled delivery, particularly for hydrophobic or lipophilic compounds. In the food sector, they can enhance nutrient absorption (e.g., vitamins, antioxidants), preserve bioactivity, and stabilize sensitive ingredients like probiotics or prebiotics. Additionally, they enable controlled flavor release, improve additive stability, and offer functionality in advanced food packaging through better barrier properties and reduced environmental footprint.

Overall, the surface-engineered alginate–halloysite biocomposites developed in this study present a promising and versatile platform for the sustained delivery of lipophilic bioactive compounds, paving the way for innovative applications in nutraceutical, pharmaceutical, and biomedical technologies.

## Figures and Tables

**Table 1:** Comparison of adsorption capacity for vitamin D_3_ with various adsorbents.

Adsorbent System	Adsorbed Compound	Adsorption Capacity (mg/g)	Reference
This study: Hydrophobically modified alginate–HNT composite	Vitamin D_3_	3.0 mg/g	—
IRMOF-3@MLDH (magnetic MOF-LDH composite)	Vitamin D_3_	126 mg/g	[58]
Polyamide-6 nanofiber scaffold with hydroxyapatite	Vitamin D_3_	0.0103 mg/g (steady state)	[59]
Magnetic graphene–sporopollenin sorbent	Vitamin D_3_	Not specified (recovery: 71.8–113.3%)	[60]

**Table 2:** Model constants and R^2^ values for vitamin D_3_ adsorption on MBHA and MHHA biocomposites: (**a**) two-parameter models, (**b**) three-parameter models.

(a)	Langmuir Model						Freundlich Model					
	q_max_			K_L_	R^2^		K_F_		n		R^2^	
MBHA	5318.108			0.48	0.9916		1611.169		1.4		0.9891	
MHHA	5935.263			0.23	0.9980		1048.538		1.33		0.9975	
**(b)**	**Sips Model**			**Redlich–Peterson Model**					**Toth Model**			
	**q_ms_**	**a_s_**	**n**	**R^2^**	**K_R_**	**a_R_**	**g**	**R^2^**	**q_mt_**	**K_T_**	**T**	**R^2^**
MBHA	3795	0.949	0.7	0.86	3100	0.5	0.69	0.98	8540	0.31	0.22	0.95
MHHA	3290	0.65	0.6	0.79	2270	0.1	0.87	0.95	3500	0.45	0.5	0.98

**Table 3:** Langmuir model comparison of MBHA and MHHA.

	q_max_	K_L_	R^2^	RSS	AIC	BIC	Chi-Square
MBHA	5318.108	0.48	0.9916	5632.43	52.3	52.51	2.39
MHHA	5935.263	0.23	0.998	2071.06	47.89	48.1	0.92

**Table 4:** Kinetic constants of vitamin D_3_ adsorption on biocomposites.

	First-Order Model			Second-Order Model		
	q_e_	K_1_	R^2^	q_e_	K_2_	R^2^
MBHA	889.5	0.011285	0.9733	1815.049	0.000587	0.999
MHHA	1130	0.018885	0.94	1855.893	0.000276	0.9969

## Data Availability

Data are contained within the article.

## Abbreviations

EEs	Encapsulation efficiency
FTIR	Fourier transform infrared spectroscopy
HTAB	Hexadecyltrimethylammonium bromide
HNTs	Halloysite nanotube nanoparticles
NPsR^2^	Correlation coefficient
UVB	Ultraviolet B
UV-Vis	UV–visible spectrophotometer
PDDA	Poly(diallyldimethylammonium) chloride
SEM	Scanning electron microscopy
SPEX	Small capacity mills
Vitamin D	D (1α25 (OH)2D) (1,25 dihydroxyvitamin D)
